# Molecular and gene expression analyses of chicken oncomodulin and their association with breast myopathies in broilers

**DOI:** 10.1016/j.psj.2024.103862

**Published:** 2024-05-21

**Authors:** Byungwhi Kong, Majid Shakeri, Janghan Choi, Hong Zhuang, Brian Bowker

**Affiliations:** USDA, Agricultural Research Service, U.S. National Poultry Research Center, Quality & Safety Assessment Research Unit, Athens, GA, USA

**Keywords:** oncomodulin, alpha-parvalbumin, Ca^2+^ signaling, chicken breast myopathy

## Abstract

Oncomodulins (**OCMs**), also known as non-α-parvalbumins, are small molecules known for their high-affinity binding of Ca^2+^ ions. They play crucial roles as Ca^2+^ buffers and participate in signaling pathways within muscle and neuron cells. In chickens, 3 oncomodulin molecules have been identified at the protein level and are named chicken oncomodulin 1 (**OCM1**), -3 (**OCM3**), and alpha-parvalbumin (**PVALB**). OCM4 was newly assigned by genome annotation. A gene cluster containing OCM1, OCM3, and OCM4 is located in chromosome 14, while a single gene of PVALB is on chromosome 1. The Ca^2+^ signaling pathway may be a potential contributor to the onset of chicken breast myopathies. However, chicken OCMs have not been extensively studied in muscle tissues. In this study, the genetic specifications, tissue-specific and differential expression of OCM1, OCM3, OCM4, and PVALB in the context of chicken breast myopathies were investigated. OCM1 exhibited moderate expression in the liver, intestine, and kidney. OCM3 was highly expressed in thymus and breast muscle. A long noncoding RNA (**lncRNA**) transcribed from the antisense strand of the OCM3 gene was found to be expressed in liver, lung, heart, intestine, and kidney tissues. OCM4 was barely expressed in thymus, thigh-, and breast muscle. PVALB exhibited high expression across all tissues examined. Results of quantitative PCR (**qPCR**) indicated that the expression of OCM3 was significantly increased (4.4 ± 0.7 fold; *P*-value = 0.03) in woody breast (**WB**) muscle and even greater (8.5 ± 0.6 fold; *P*-value = 0.004) in WB/white striping (**WS**) muscles. The expression of PVALB showed no difference in WB muscle, but it was notably higher (4.6 ± 0.7 fold; *P*-value = 0.054) in WB/WS muscle, although statistical significance was not reached. These findings suggest that increased expression of OCM3 and PVALB may be linked to chicken breast myopathies with regard to disruption of Ca^2+^ buffering.

## INTRODUCTION

Oncomodulins (**OCMs**), also known as non-α-parvalbumins, are small proteins typically composed of 108 to 109 amino acids (Dijkstra and Kondo, 2022). They play pivotal roles in Ca^2+^ signaling (Verkhratsky and Parpura, 2014). In eukaryotic signal transduction pathways, Ca^2+^ functions as a secondary messenger and is primarily regulated by EF-hand proteins characterized by 2 α-helices (N-terminal ‘E’ helix and C-terminal ‘F’ helix) connected by a Ca^2+^-binding loop (Kawasaki et al., 1998). OCMs are a type of vertebrate-specific EF-hand protein belonging to the calmodulin superfamily (Girard et al., 2015). The name “oncomodulin” stems from the discovery of OCMs in certain mammalian tumors (MacManus, 1979).

In mammals, OCMs are cytosolic proteins found in fast-twitch muscle fibers, parathyroid glands, and the distal convoluted tubules of nephrons (Celio and Heizmann, 1982; Pauls et al., 2000; Olinger et al., 2012; Henzi and Schwaller, 2015). In chickens, 3 distinct OCMs, known as OCM1, OCM3, and α-parvalbumin (**PVALB**), were identified at the protein level (Brewer et al., 1989; Hapak et al., 1994; Henzl et al., 1991; Kuster et al., 1991). They have been referred to as chicken parvalbumins or chicken thymic hormones. A recent additional OCM, OCM4, was identified through computational annotation of the chicken genome (https://www.ncbi.nlm.nih.gov/gene/427654). OCM1 and -3 are known as thymic proteins, which are expressed in stromal cells (Brewer et al., 1989; Henzl et al., 1991; Kuster et al., 1991; Hapak et al., 1994), in addition to eyes and hair cells in auditory organs (Brewer et al., 1991; Heller et al., 2002; Rada and Wiechmann, 2009). OCM3 shares similarities with parvalbumins found in amphibians and reptiles (Brewer et al., 1991; Rada and Wiechmann, 2009), but a direct mammalian homologue of OCM3 is absent. Instead, chicken OCM1 is considered to be a mammalian homologue of OCMs. PVALB was identified as a muscle-specific member of the OCM family (Brewer et al., 1991; Kuster et al., 1991). It is a cytosolic protein capable of binding to Ca^2+^ ions and may play a role in maintaining Ca^2+^ homeostasis within the sarcoplasmic reticulum of muscle cells (Raymackers et al., 2000). A recent study revealed elevated PVALB mRNA expression in WB myopathy compared to non-diseased control breast muscle (Mutryn et al., 2015).

Chicken breast myopathies, which include conditions like WB (characterized by muscle hardness), WS (characterized by white striations on breast meat surface), and spaghetti meat (muscle bundle separation resulting in noodle-like appearance in breast meat), have led to significant economic losses in the US and global poultry production sectors. Although the exact causes of these conditions remain unclear, disrupted Ca^2+^ homeostasis may play a role in their onset [reviewed in (Soglia et al., 2021)]. Both increased accumulation of reactive oxygen species (**ROS**) and heightened stress in the sarcoplasmic reticulum appear to impact Ca^2+^ homeostasis by raising Ca^2+^ concentration in myopathic breast muscles (Zambonelli et al., 2016; Kropski and Blackwell, 2018). The intracellular Ca^2+^ overload could potentially alter the sarcolemmal integrity contributing to the development of breast myopathic phenotypes (Sandercock and Mitchell, 2004). In addition to the observed upregulation of PVALB gene expression in WB muscle (Mutryn et al., 2015), another global gene expression study also indicated a decrease in the expression of a newly computationally assigned chicken OCM4 (**EMBL** accession: ENSGALG00000021286) in early pathogenic WB muscle in broilers at 3 wk of age (Papah et al., 2018). Given this background information, it was hypothesized that chicken OCMs and PVALB may be involved in the disruption of Ca^2+^ concentration observed in chicken breast myopathies. The present study aimed to investigate the genetic structure, tissue-specific expression, and the expression patterns of OCM1, OCM3, OCM4, and PVALB in the context of chicken breast myopathies.

## MATERIALS AND METHODS

### Gene Information and Amino Acid Sequence Analysis

Gene information for OCM and other genes was obtained from National Center for Biotechnology Information (**NCBI**) database (https://www.ncbi.nlm.nih.gov/). Accession numbers for each OCM isoform, as well as for lncRNA, are presented in Table 1. Multiple sequence alignment and phylogenetic tree analysis of OCM isoforms were performed using the online Clustal Omega program provided by the European Bioinformatics Institute (EMBL-EBI) (https://www.ebi.ac.uk/Tools/msa/clustalo/). Amino acid percent similarities were determined by BlastP (protein Blast) function (https://blast.ncbi.nlm.nih.gov/Blast.cgi?PAGE=Proteins).

**Table 1:** Accession numbers for OCMs and a lncRNA.

Name		Gene	mRNA	Protein
OCM1	1NCBI ID	386586	NM_204422.1	XP_046783222.1
	2Length (bp, nt, or aa)	3515 bp	640 nt	109 aa
OCM3	NCBI ID	396531	NM_001007477.4	NP_001007478.1
	Length (bp, nt, or aa)	4899 bp	764 nt	109 aa
OCM4 var.1	NCBI ID	427654	XM_015294286.4	XP_025011090.1
	Length (bp, nt, or aa)	3153 bp	958 nt	130 aa
OCM4 var.2	NCBI ID	427654	XM_025155322.3	XP_015149772.2
	Length (bp, nt, or aa)	3153 bp	1054 nt	129 aa
PVALB	NCBI ID	396459	NM_001159315.2	NP_001152787.1
	Length (bp, nt, or aa)	13255 bp	2287 nt	110 aa
lncRNA (LOC112533467)	NCBI ID	112533467	XR_003077739	
	Length (bp, nt, or aa)	34111 bp	894 nt	

1 NCBI, National Center for Biotechnology Information (https://www.ncbi.nlm.nih.gov/).

2 bp, base pairs; nt, nucleotides; aa, amino acids.

### Collection of Organ Tissues From Embryo

Organ tissue samples including thymus, liver, lung, heart, kidney, intestine, thigh muscle, and breast muscle were collected from 19-day-old chicken embryos of a random-bred research chicken line. Organ tissue samples were used for determining tissue specific-gene expression of OCM isoforms. Chicken embryos were provided by Dr. Sara Orlowski at the University of Arkansas, Poultry Research Facility in Fayetteville, Arkansas, USA.

### Birds and Collection of Breast Muscles With Woody Breast and White Striping

For the gene expression of OCMs associated with chicken breast myopathies, 49-day-old Ross 708 commercial male broiler chickens were used. Ethical protocols for the care and experimental use of animals particularly for the collection of chicken breast muscle samples from normal and myopathic conditions, were approved by the University of Arkansas Institutional Animal Care and Use Committee (IACUC #: 18083). The chickens were raised in accordance with standard management practices at the Poultry Farm, University of Arkansas. Briefly, 25 Ross 708 broiler chicks were grouped and placed a floor pen (1.2 × 1.82 m; 0.09 m^2^ per bird). Pens were equipped with fresh pine shavings, a hanging feeder, and a nipple drinker water line. Birds had access to a standard commercial diet ad libitum. Internal house environment kept constant with a set point temperature of 32°C when chicks were placed. Temperature was decreased 2°C per week until reached an endpoint of 15°C. Lighting was maintained as hours of light (**L**) to hours of dark (**D**) as follows: 24L:0D from d 0 to 1, 23L:1D from d 1 to 7, and 16L:8D from d 7 to 56. Starter diets were fed as crumbles from d 0 to 14, whereas the grower, finisher, and withdrawal diets were fed as pellets from d 15 to 28, 29 to 42, and 43 to 49 d of age, respectively (Kong et al., 2024). No additional treatment was used in the diet. At d 49, chicken breast muscles were extracted followed the procedures outlined by Kang et al. (2020). Briefly, live birds underwent physical palpation for WB prediction and at less than 10 min postslaughter, WB and/or WS conditions of the breast muscles (pectoralis major) were evaluated by visual examination. Based on a scoring scale of 0 to 3 (Kang et al., 2020; Kong et al., 2021), they were classified into control (Con, n = 6; WB and WS scores = 0), WB (n = 7; WB score ≥ 2, WS score ≤ 1), and WB/WS (n = 7; both WB and WS scores ≥ 2) categories. Breast muscle samples were collected at less than 10 minutes post-mortem, immediately snap-frozen in dry ice, and stored at -80°C until further use.

### RNA Isolation, RT-PCR, qPCR, and Statistical Analyses

RNA extraction, cDNA synthesis, and PCR were performed following the methods of Kong et al. (2017). Briefly, total RNA was extracted from quick-frozen breast muscle and organ tissues using TRIzol reagent (ThermoFisher Scientific, Waltham, MA), followed by DNase I treatment (ThermoFisher Scientific, Waltham, MA), and re-purification using the RNeasy mini kit (Qiagen, Valencia, CA). RNA quality and quantity were assessed through Tape Station 4200 digital electrophoresis (Agilent Technologies, Santa Clara, CA) and NanoDrop 1000 (ThermoFisher Scientific, Waltham, MA). To synthesize cDNA, 1 µg of total RNA was used with oligo (dT)16 primer or random hexamer (for 18s rRNA only), along with SuperScript III reverse transcriptase (ThermoFisher Scientific, Waltham, MA). Oligonucleotide primers were designed using the PRIMERS3 program (http://frodo.wi.mit.edu) and were commercially synthesized by IDT Inc. (Coralville, IA). The primer list for this study is available in Table 2. The expression of OCMs in various tissues was determined using conventional reverse transcription PCR (**RT-PCR**) and Tape Station 4200 digital electrophoresis. All PCR reactions utilized EvaGreen master mix (Qiagen, Valencia, CA) with 12 µL total volume containing cDNA and each 0.4 µM forward- and reverse primers. PCR cycles include 95°C for 2 min for initial denaturation, 40 cycles of 95°C for 30 s, 60°C for 1 min, 72°C for 30 s, and 72°C for 10 min for the last extension. The 2 µL of PCR products were mixed with 2 µL sample buffer of D1000 Screen Tape (Agilent Technologies, Santa Clara, CA), the mixture was electrophorized in the capillaries of the D1000 Screen Tape, and digitalized images were captured with Tape Station software. Quantitative PCR was used to evaluate OCM expression in chicken breast muscles using the Quant Studio 6 Flex system (ThermoFisher Scientific, Waltham, MA). The expression of 18S rRNA was assessed using cDNAs synthesized separately with random hexamers, rather than oligo dT primers. Relative expression was calculated using the 2^−ΔΔCt^ method, statistical analysis was performed using the t-test to compare myopathic muscle samples (WB or WB/WS) with control muscle, and the level of statistical significance was set to *P*-value < 0.05.

**Table 2:** Primers used for reverse transcription PCR (RT-PCR).

Name	Sequences	Product size (bp)
18s_F	TCCCCTCCCGTTACTTGGAT	60
18s_R	GCGCTCGTCGGCATGTA	
GAPDH_F	CCGTGTTGTGGACTTGATGG	92
GAPDH_R	CAACAAAGGGTCCTGCTTCC	
OCM4_X1 _F	ATGATGGA GGAGATCAATACTAACT	393
OCM4_X1_R	TTAAGATTTGACCAGGGCTTGAAACT	
OCM4_X2_F	GTGCTTGGCTTGGGAAAACT	113
OCM4_X2_R	AAGAGGAGGCTGTTCTGTCC	
OCM4_all_F	GGGTCTGAACTCCAAGTCCA	181
OCM4_all_R	TCCATCACTGTCTCCTGCTG	
OCM1_F	TGCCAAGCTCCAGATTCCTT	176
OCM1_R	ACTCTGGCTCCACACTCAAA	
OCM3_F	GGCTGCGGATTCCTTCAATT	199
OCM3_R	GAAAGCTTTGGTCTCCGCAG	
PVLAB_F	AGCCCAGAAGACGTGAAGAA	197
PVLAB_R	GTTGCAAATTCATCAGCGCC	

## RESULTS AND DISCUSSION

### Analysis of Chicken OCMs: Gene Information, Amino Acid Sequences, and Sequence Similarities

**Figure 1:**
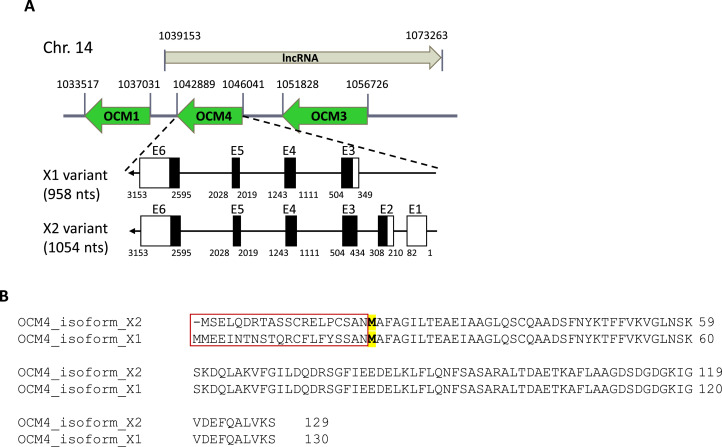
Gene structures of chicken OCMs in chromosome 14. (A) The numbers indicate genomic locations. Arrows show the direction of gene expression. Boxed regions indicate exons (E1–E6), while lined regions show introns for OCM4 gene. Closed (black) boxes indicate coding DNA sequences (CDS) and open (white) boxes show untranslated regions of mRNA. A region for expressing a long noncoding RNA (lncRNA) was placed in the figures. (B) Amino acid sequences of X2 and X1 variants are aligned, with each position shown. The red box indicates a hyper-variable region. The second internal translation initiation codon was marked in yellow and bolded.

Gene, mRNA, and protein information for all OCMs were retrieved from NCBI database. Notably, the gene cluster for OCM1, OCM3, and OCM4 is located in Chromosome 14 in the antisense direction (Figure 1), whereas a single PVALB gene is situated in Chromosome 1 in the sense direction (not depicted here). Interestingly, in the opposite direction to the OCM gene cluster, a lncRNA (LOC112533467) was identified. Accession numbers and genomic DNA, mRNA, and amino acid sequence lengths for OCMs (not for lncRNA) are documented in Table 1. It is worth noting that OCM4 presents 2 transcript variants (X1 and X2) with distinct exon combinations and alternative translation initiation codons. OCM4X1 is comprised of exons E3 to E6, while OCM4X2 is linked to exons E1 to E6 (Figure 1). Although the N-terminal ∼20 amino acids of these 2 variants differ (highlighted in the red box in Figure 1B), the last 109 amino acids, starting from an internal methionine residue (bold and highlighted in Figure 1B), remain identical between the 2 variants.

**Figure 2:**
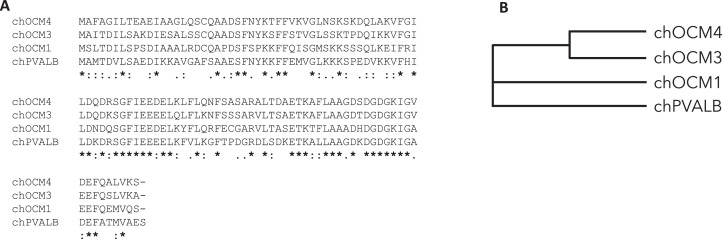
Amino acid sequence comparisons among OCM isoforms and PVALB. (A) Alignment of amino acid sequences for chOCM4, chOCM3, chOCM1, and chPVALB. Each mark of amino acid conservation is indicated as follow: * denotes “conserved”; : (colon) signifies “strongly similar property”; . (period) represents “weakly similar property”; Blank indicates “dissimilar property.” (B) Phylogram illustrates similarities among chicken OCMs.

Figure 2 shows the amino acid alignment (Figure 2A) and the phylogram (Figure 2B) among OCM1, OCM3, OCM4 (homologous regions between X1 and X2 variants) and PVALB proteins. Phylogram results revealed that OCM3 and OCM4 were the closest, while OCM1 and PVALB were the farthest (Figure 2B). Blast search results showed that chicken OCMs exhibit a range of 56 to 72.5% similarity, suggesting moderate conservation among them (Table 3).

**Table 3:** Percent identities are presented among chicken OCM isoforms.

	OCM1	OCM3	OCM4	PVALB
OCM1	100			
OCM3	59.6	100		
OCM4	58.7	72.5	100	
PVALB	53.3	60.8	56	100

### mRNA Expression of OCMs and PVALB in Various Tissues

**Figure 3:**
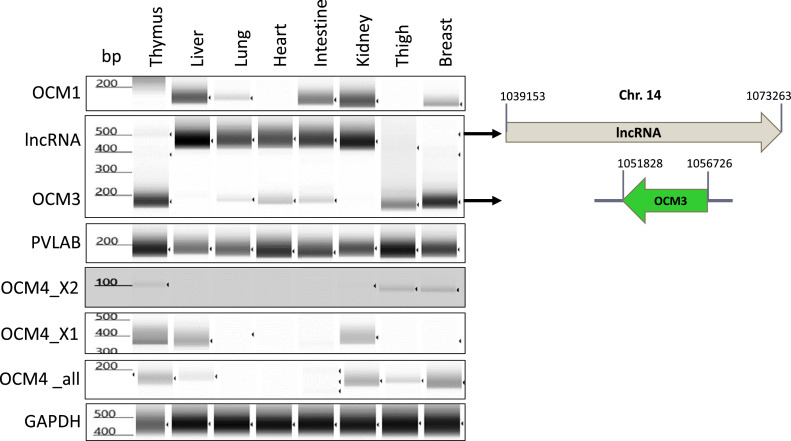
mRNA expression of OCM isoforms, PVALB, and a lncRNA in various organ tissues. The “bp” notation indicates a base-pair, serving as a DNA length marker. GAPDH is used as a loading control. PCR amplicon for both lncRNA and OCM3 is indicated in right side of the gel image. OCM4_all, which were amplified with primer pairs of OCM4_all_F and OCM4_all_R (Table 2), indicates PCR products amplifying the homologous RNA sequences found at 3′ flanking region of the 2nd AUG codons (Methionine highlighted in Figure 1B) in both OCM4_X1 and X2 variants.

To compare the organ tissue-specific expression patterns among various tissues with breast muscle, which may be linked to functionalities of isoforms, mRNA expression of OCMs and PVALB was determined by reverse transcription PCR using RNA extracted from thymus, liver, lung, heart, intestine, kidney, thigh muscle, and breast muscle (Figure 3). OCM1 was predominantly expressed in the liver, intestine, and kidney, with moderate expression in the lung and breast muscle. However, it was not detected in the thymus, heart, and thigh muscles. OCM3 exhibited high expression in the thymus and breast muscle, along with moderate expression in the thigh muscle. Interestingly, additional cDNAs were amplified during PCR, revealing the presence of a long non-coding RNA (lncRNA; LOC112533467). This lncRNA is expressed in the opposite direction of the OCM gene cluster in Chromosome 14 (Figure 1), and it was found in the liver, lung, heart, intestine, and kidney, where OCM3 was not highly expressed. Typically, lncRNAs are known to regulate gene expression, mostly down-regulating it, and can influence transcriptional, post-transcriptional, and translational processes (Statello et al., 2021). Thus, the expression of this lncRNA might suppress OCM3 expression (or mRNA stability) in specific organ tissues. The clear separation between the tissue-specific expression of OCM3 (in the thymus and breast muscle) and the lncRNA (not present in the thymus and breast muscle) can explain why OCM3 functions are limited to specific tissues, such as the thymus and breast muscles. PVALB exhibited high expression in all the tissues examined in this study, indicating that PVALB may play an essential role in regulating homeostasis in all organ tissue environments. Regarding OMC4, reverse transcription PCR was conducted using specific forward and reverse primers for the X1 or X2 variant. Additionally, a set of primers was used to amplify the common region between X1 and X2 variants to assess overall OCM4 expression. The results indicate that OCM4X2 variant is expressed in the thymus, thigh muscle, and breast muscle, while X1 variant is present in the thymus, liver, and kidney but not in skeletal muscles (Figure 3). Expression levels for both OCM4 variants are notably lower in breast muscle compared to those of OCM3 and PVALB. Correspondingly, the PCR results for the common region show similarly low expression levels, consistent with the results of the variant-specific PCR amplifications (Figure 3). OCM4 was computationally assigned through the chicken genome sequencing project and has yet to be confirmed by actual mRNA or protein expression. OCM4 expression levels were confirmed by aligning OCM4 mRNA sequences with raw reads data of RNA sequencing studies on breast muscle and liver retrieved from the NCBI and authors’ databases. Only a few sequence reads were aligned with OCM4 mRNA sequences, but they did not cover full coding DNA sequences (CDS; protein coding region) (data not shown). Thus, OCM3 and PVLAB may be dominant isoforms in chicken skeletal muscle compared to OCM1 and OCM4.

### Differential Expression of OCMs in Breast Myopathies

**Figure 4:**
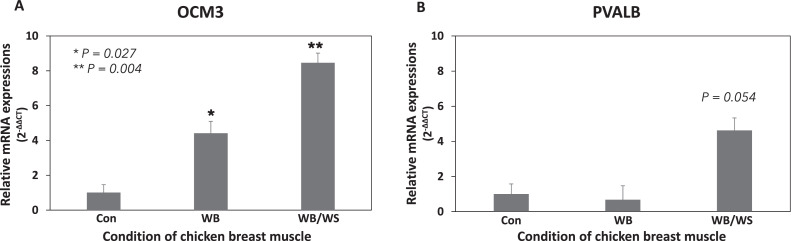
Gene expression of OCM3 (A) and PVALB (B) in chicken breast muscles with non-diseased control (**Con**), woody breast myopathy (**WB**), and woody breast/white striping myopathies (WB/WS). Gene expression analysis was performed using quantitative PCR. The fold change values (2^−ΔΔCT^) on the y-axis are based on Con samples, with * denoting *P* < 0.05 and ** indicating *P* < 0.01.

Breast muscles affected by degenerative diseases, including WB and WS, display disruptions in Ca^2+^ buffering conditions (Zhang et al., 2023b). OCMs showed significant expression in chicken breast muscle. Thus, the expression of OCMs were assessed in breast myopathy samples. mRNA expression of OCM3 demonstrated a significant increase (4.4 ± 0.7 fold; *P*-value = 0.03) in WB muscle and an even greater increase (8.5 ± 0.6 fold; *P*-value = 0.004) in WB/WS muscles compared to nondiseased control muscles (Con) (Figure 4A). PVALB expression remained unchanged in WB and tended to be higher (4.6 ± 0.7 fold; *P*-value = 0.054) in WB/WS samples compared to Con (Figure 4B). Expression of OCM1 and OCM4 was very low and did not exhibit differences in myopathic samples (data not shown). These results suggest that the increased expression of OCM3 may be a mechanism to counterbalance Ca^2+^ imbalances in breast myopathies. OCM3 appears to be activated in both WB and WS conditions, while PVALB may be activated only in WS conditions, but not in muscle exhibiting only the WB myopathy.

Previous studies using transcriptomics, proteomics, and metabolomics, showing differentially expressed/abundant genes, proteins, and metabolites have proposed contributing factors to Ca^2+^ concentration disruptions in the myopathic breast phenotypes [reviewed in (Zhang et al., 2023a)]. Mammalian cytosolic parvalbumin (homologue of chicken PVALB), when bound to Ca^2+^, may contribute to the shortening contraction-relaxation cycle in fast-twitch muscle fibers of rodents (Raymackers et al., 2000). Thus, the transcriptional upregulation of PVALB in WB/WS breast muscles was considered to be a compensatory change aimed at buffering Ca^2+^ ions and preventing hypercontraction of the fibers (Mutryn et al., 2015). Additionally, WB myopathic muscle showed a higher proportion of hypercontracted myofibers (Sihvo et al., 2014), and a significant increase in Ca^2+^ concentration compared to nondiseased control breast muscle (Soglia et al., 2016; Tasoniero et al., 2016). However, as noted earlier, PVALB expression was not increased in WB only myopathic samples but tended to increase in WB/WS co-myopathic samples, suggesting that PVALB may respond primarily to WS conditions with issues in structural integrity of fiber striations.

## CONCLUSIONS

In this study, the genetic structure, and gene expression patterns of chicken OCM isoforms and PVALB were explored with chicken breast myopathies as well as with chicken organs. The disruption of Ca^2+^ homeostasis in the sarcoplasmic reticulum has been considered a potential cause of the onset of chicken breast myopathies. This study suggests that increased expression of OCM3 and PVALB could be involved in the incidence of chicken breast myopathies showing Ca^2+^ dyshomeostasis, and further research is warranted to characterize the functions of OCMs in alleviating chicken breast myopathies.

## DISCLOSURES

The authors declare no conflicts of interest.
